# Supersymmetric fits after the Higgs discovery and implications for model building

**DOI:** 10.1140/epjc/s10052-014-2732-7

**Published:** 2014-05-27

**Authors:** John Ellis

**Affiliations:** 1 Department of Physics, King’s College London, London, WC2R 2LS UK; 2 Theory Division, CERN, 1211 Geneva 23, Switzerland

## Abstract

The data from the first run of the LHC at 7 and 8 TeV, together with the information provided by other experiments such as precision electroweak measurements, flavour measurements, the cosmological density of cold dark matter and the direct search for the scattering of dark matter particles in the LUX experiment, provide important constraints on supersymmetric models. Important information is provided by the ATLAS and CMS measurements of the mass of the Higgs boson, as well as the negative results of searches at the LHC for events with $E{/}_T$ accompanied by jets, and the LHCb and CMS measurements of $\mathrm{BR}(B_s \rightarrow \mu ^+\mu ^-)$. Results are presented from frequentist analyses of the parameter spaces of the CMSSM and NUHM1. The global $\chi ^2$ functions for the supersymmetric models vary slowly over most of the parameter spaces allowed by the Higgs mass and the $E{/}_T$ search, with best-fit values that are comparable to the $\chi ^2$ for the standard model. The 95 % CL lower limits on the masses of gluinos and squarks allow significant prospects for observing them during the LHC runs at higher energies.

## Introduction

The discovery of a Higgs boson at the LHC [1, 2] has given new heart to advocates of supersymmetry [3]. Its mass is consistent with the predictions of minimal supersymmetric models that the lightest Higgs boson should weigh $\lesssim$130 GeV [4–11]. Indeed, the measured value of $m_h$ lies in the range where new physics seems to be required to stabilize the electroweak vacuum [12], which might well be supersymmetry [13]. Moreover, the measurements of Higgs couplings to other particles are consistent with the predictions of many supersymmetric models, which are close to those in the standard model. There are no signs so far of the deviations from the standard model couplings that are characteristic of models in which electroweak symmetry breaking is driven by some new dynamics [14].

On the other hand, neither are there any signs for other types of new physics, such as might be responsible for dark matter in the form of massive, weakly interacting particles whose production could be inferred in searches for events with jets and missing transverse energy, $E{/}_T$ at the LHC. Supersymmetry with conserved $R$ parity is one such model that suggests the existence of a dark matter particle that was in thermal equilibrium in the early Universe and should weigh $\sim 1$ TeV if it is to have the appropriate cosmological relic density [15]. It is assumed here that the lightest supersymmetric particle (LSP) that constitutes the dark matter is the lightest neutralino $\chi$ [16, 17], though there are other candidates such as the gravitino. Important constraints on such dark matter models are imposed by direct and indirect searches for dark matter, as well as by LHC searches for $E{/}_T$ events, none of which have found convincing signals [18].

Even if $R$ conservation is assumed, the interpretation of all these constraints is quite model dependent. For simplicity, we consider here only the minimal supersymmetric extension of the standard model (the MSSM), though there are well-motivated extensions, e.g., to include any extra singlet superfield (the NMSSM [19]). The MSSM already has over 100 parameters, and it is natural to consider simplifying hypotheses such as minimal flavour violation (MFV), in which all flavour violation is related to Cabibbo–Kobayashi–Maskawa mixing [20, 21]. In principle, this model has six additional CP-violating phases [22], but upper limits on electric dipole moments offer no suggestion that they are large. Many studies of experimental constraints focus on versions of the MSSM with MFV in which the soft supersymmetry-breaking contributions to sfermion, Higgs and gaugino masses, $m_0$ and $m_{1/2}$, respectively, as well as trilinear couplings $A_0$, are constrained to be universal at some high input scale (the CMSSM) [23–32], or in generalizations in which the soft supersymmetry-breaking contributions to Higgs masses are allowed to be non-universal but equal (the NUHM1) [33–35]. One example of a more restrictive model is minimal supergravity (mSUGRA), in which the gravitino mass is forced to be equal to the input scalar mass: $m_{3/2} = m_0$, and the trilinear and bilinear soft supersymmetry-breaking parameters are related: $A_0 = B_0 + m_0$.

As we shall see, the LHC $$E{/}_T$$ searches impose strong constraints on models with universal soft supersymmetry-breaking parameters such as the CMSSM, NUHM1 and mSUGRA, stimulating interest in ‘natural’ models in which the third-generation squarks are much lighter than those of the first and second generations, for which experiments give weaker constraints. Also, searches for specific $$E{/}_T+ $$ jets signatures have been interpreted within simplified models in which these topologies are assumed to be the dominant supersymmetric signatures. There has also been interest in using searches for $$E{/}_T+ $$ monojet, monophoton and mono-$$W/Z$$ topologies to look for the direct pair-production of dark matter particles without passing via the cascade decays of heavier sparticles.

In view of its importance for constraining supersymmetric models, in Sect. 2 of this review there is a discussion of Higgs mass calculations and their uncertainties, as well as indications of their implications for the parameter spaces of supersymmetric models. Section 3 presents some results of global fits [36] to the CMSSM and NUHM1 using the full $E{/}_T$ data from Run 1 of the LHC at 7 and 8 TeV [37], the measurement by CMS and LHCb of $\mathrm{BR}(B_s \rightarrow \mu ^+\mu ^-)$ [38–42], and the latest constraints on dark matter scattering from the LUX experiment [43]. These results include 95 % CL lower limits on sparticle masses and the prospects for discovering them in Run 2 of the LHC at 13/14 TeV. Section 4 summarizes some pertinent results within other frameworks such as mSUGRA, ‘natural’ and simplified models. Finally, Sect. 5 draws some conclusions for supersymmetric model-building.

## The Higgs mass and supersymmetry

As is well known, the two complex Higgs doublets of the MSSM have eight degrees of freedom, of which three give masses to the $W^\pm$ bosons and to the $Z^0$ via the electroweak symmetry breaking, leaving five physical Higgs bosons in the physical spectrum: two neutral Higgs bosons $h, H$ that are CP-even (scalar), one neutral boson $A$ that is CP-odd (pseudoscalar), and two charged bosons $H^{\pm }$. The tree-level masses of the scalar supersymmetric Higgs bosons are

1 $$\begin{aligned} m^2_{h, H} \!=\! \frac{1}{2}\left( m_{A}^2\!+\!m_Z^2 \mp \sqrt{(m_{A}^2\!+\!m_Z^2)^2 \!-\!4m_{A}^2m_Z^2\cos ^2 2\beta }\right) \nonumber \\ \end{aligned}$$

where $\tan \beta$ is the ratio of Higgs v.e.v.s, from which we see that $m_h$ is bounded from above by $m_Z$. 1 However, there are important radiative corrections to $m_h$ (1) [4–11], of which the most important is the one-loop correction due to the top quark and stop squark:

2 $$\begin{aligned} \Delta m_h^2=\frac{3m_t^4}{4\pi ^2 v^2}\ln \left( \frac{m_{\tilde{t}_1}m_{\tilde{t}_2}}{m_t^2}\right) + \cdots , \end{aligned}$$

where $m_{\tilde{t}_{1,2}}$ are the physical masses of the stops. We see in (2) that the correction $\Delta m_h^2$ depends quartically on the mass of the top, and it implies that the mass of the lightest Higgs boson may be as large as

3 $$\begin{aligned} m_h \lesssim 130~\mathrm{GeV}. \end{aligned}$$

for stop masses of about a TeV, consistent with the ATLAS and CMS measurements [1, 2].

**Fig. 1:**
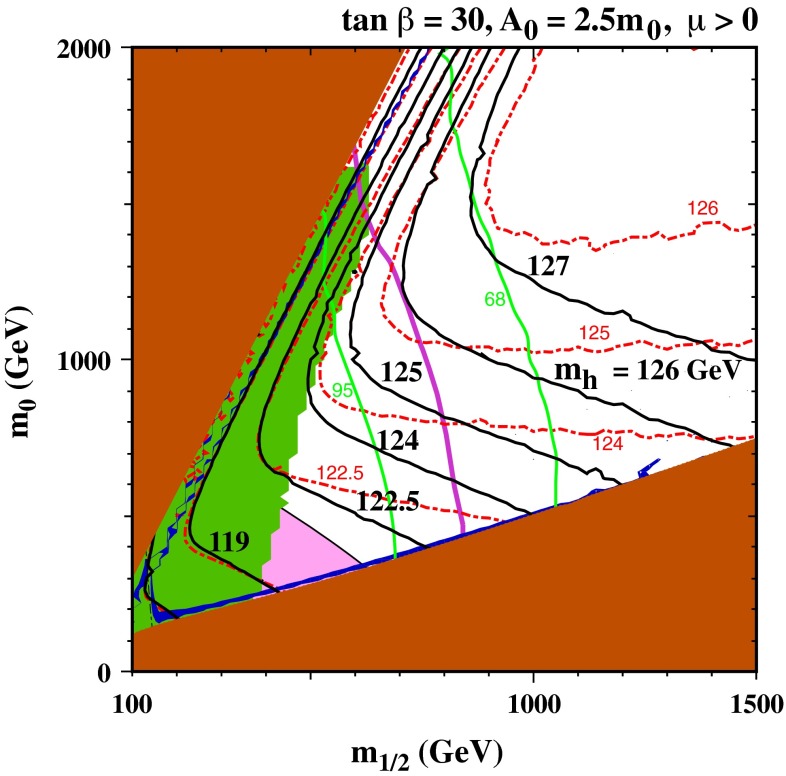
The allowed regions in the $(m_{1/2}, m_0)$ plane for $\tan \beta = 30$ and $A_0 = 2.5 m_0$ [44]. The *line styles* and *shadings* are described in the text. The section of the dark blue coannihilation strip in the range $m_{1/2} \in (840, 1{,}050)$ GeV is compatible with the constraints from BR($B_s \rightarrow \mu ^+ \mu ^-$) (*green lines* marking the 68 and 95 % CL) [38–42] and the ATLAS 20/fb MET search (*purple line*) [37], as well as with the LHC $m_H$ measurement. Good consistency with all the constraints is found if the improved FeynHiggs 2.10.0 code [45–50] is used (*black lines*): results from a previous version of FeynHiggs are indicated by *red dotted lines*

If one wishes to use (2) to estimate the stop mass scale, it is clear that the answer is exponentially sensitive to the Higgs mass, and it is therefore important to refine the one-loop calculation. Several codes are available that provide complete two-loop calculations and include the leading dependences of three- and higher-loop contributions on the strong coupling $\alpha _s$ and the top Yukawa coupling $\alpha _t$. It is also important to estimate the theoretical uncertainty in the calculation of $m_h$ for given values of the supersymmetric model parameters, which is typically $\sim 1.5$ to 3 GeV. In the following, results from the FeynHiggs 2.10.0 code for calculating $m_h$ are used, which is a significant improvement over previous versions. As an example of the importance for inferences about the supersymmetric mass scale from the measured value of $m_h$, Fig. 1 displays the $(m_{1/2}, m_0)$ plane in the CMSSM for $\tan \beta = 30$, $\mu > 0$ and $A_0 = 2.5 m_0$ [44].

The brown shaded wedge at large $m_{1/2}$ and small $m_0$ is excluded because there the LSP would be the charged ${\tilde{\tau }_1}$, whereas the lighter stop, ${\tilde{t}_1}$, would be the LSP. Adjacent to these wedges are narrow blue strips where the relic LSP density falls within the range favoured by astrophysics and cosmology. Measurements of $b \rightarrow s \gamma$ exclude the region shaded green, whereas in the pink region the discrepancy between the standard model and experimental values of the anomalous magnetic moment of the muon, $g_\mu - 2$, could be explained by supersymmetry [51]. The 95 % CL limit on $E{/}_T$ + jets events at the LHC [37] is represented by the purple line, and the green lines represent 68 and 95 % CL limits from the value of $\mathrm{BR}(B_s \rightarrow \mu ^+\mu ^-)$ measured by the CMS and LHCb experiments [38–42]. Finally, the black lines are contours of $m_h$ calculated with the current version 2.10.0 of the FeynHiggs code [45–50], which includes the leading and next-to-leading $\log (m_{\tilde{t}}/m_t)$ terms in all orders of perturbation theory, as calculated using the two-loop renormalization-group equations (RGEs). The red dashed lines are calculated with an earlier version of FeynHiggs, which did not include these refinements, and we see that the $m_h$ contours diverge significantly at large $m_{1/2}$, in particular. We also see that there is a region with $(m_{1/2}, m_0) \sim (1{,}200, 600)$ GeV that is compatible with dark matter and laboratory constraints (except for $g_\mu - 2$) and corresponds to $m_h \sim 125$ GeV according to the latest version of FeynHiggs, whereas the earlier version would have yielded $m_h < 124$ GeV [44].

Smaller values of $\tan \beta$ would yield smaller values of $m_h$, and larger values of $\tan \beta$ would be more tightly constrained by $\mathrm{BR}(B_s \rightarrow \mu ^+\mu ^-)$, though values of $\tan \beta \lesssim 50$ may be compatible with all the constraints. Smaller values of $A_0$ would also yield smaller values of $m_h$ along the strip near the boundary of the ${\tilde{\tau }_1}$ LSP wedge where the appropriate dark matter density is obtained, and this dark matter strip would only extend to lower $m_{1/2}$ in this case. There is a second dark matter strip close to the boundary with the ${\tilde{t}_1}$ LSP region, but $m_h$ is too small except possibly at very large values of $m_0$ [44]. In general, CMSSM models with an LHC-compatible value of $m_h$ do not make a significant contribution to resolving the $g_\mu - 2$ discrepancy [51].

## Global fits in the CMSSM and NUHM1

After this first taste of the interplay between the LHC $E{/}_T$, $m_h$, $\mathrm{BR}(B_s \rightarrow \mu ^+\mu ^-)$, dark matter and other constraints, and their potential implications for models, I now present some results from a global fit to the relevant data within the CMSSM [36]. These are compared with the results of a fit within the NUHM1, which offers, in principle, new ways to reconcile some of the constraints discussed in the previous section.

These fits are based on a frequentist approach developed by the MasterCode collaboration [52–69], and the MultiNest tool is used to sample the CMSSM and NUHM1 parameter spaces [70–72]. The global $\chi ^2$ function is calculated including precision electroweak observables such as $M_W$ and measurements at the $Z^0$ peak, as well as $g_\mu - 2$. Also included is a full suite of flavour observables such as $b \rightarrow s \gamma$ and $B \rightarrow \tau \nu$ as well as $\mathrm{BR}(B_s \rightarrow \mu ^+\mu ^-)$  [36]. In addition to the dark matter density, a contribution from the LUX direct search [43] for the scattering of astrophysical dark matter is also included.

Figure 2 displays $(m_0, m_{1/2})$ planes in the CMSSM (left panel) and the NUHM1 (right panel), both with $\mu > 0$.^2^ The best-fit points are indicated by green stars, the $\Delta \chi ^2 = 2.30$ contours, which correspond approximately to the 68 % CL are shown as red lines, and the $\Delta \chi ^2 = 5.99$ contours, which correspond approximately to the 95 % CL are shown as blue lines. The results of the current fit [36] are indicated by solid lines and solid stars, whilst the dashed lines and open stars represent the results of fits to the data used in [61], reanalyzed using the current version of MasterCode.

In both the CMSSM and the NUHM1, we see two distinct regions: a smaller region around $(m_0, m_{1/2}) \sim (500, 1{,}000)$ GeV and a larger region extending to larger values of $(m_0, m_{1/2}$. The low-mass regions correspond to the ${\tilde{\tau }_1}$ coannihilation strip mentioned in the previous section, and in the high-mass regions other mechanisms bring the relic LSP density into the range allowed by astrophysics and cosmology, notably rapid LSP annihilation via direct-channel $H/A$ resonances when $m_\chi \sim m_{H/A}/2$, and neutralino–chargino coannihilation, which becomes more important when the LSP has a significant Higgsino component. The extra parameter in the NUHM1 Higgs sectors offers more possibilities for these effects, enabling the relic density constraint to satisfied at larger values of $m_{1/2}$ and smaller values of $\tan \beta$ than in the CMSSM [44].

As we see in Table 1, the minimum values of $\chi ^2$ in the low- and high-mass regions differ by less than unity in both the CMSSM and the NUHM1. In the case of the CMSSM, the contribution from $g_\mu - 2$ is smaller in the low-mass region, but the contribution from the ATLAS jets + $E{/}_T$ search is larger. This is also the case in the NUHM1, but other observables such as $A_{fb}(b)$ and $A_\ell (\mathrm{SLD})$ also contribute differences in $\chi ^2$ between the low- and high-mass regions that are $\mathcal{O}(1)$ [36]. In general, the global $\chi ^2$ function varies little over much of the $(m_0, m_{1/2})$ planes explored. Also, the value of $\chi ^2$ at the global minimum in the CMSSM is not significantly different from that in the standard model, whereas that in the NUHM1 is $\sim 2$ lower [36]. The CMSSM and NUHM1 confer no convincing advantages over the standard model in the global fits reported here.

**Table 1:** The best-fit points found in global CMSSM and NUHM1 fits with $\mu > 0$, using the ATLAS $E{/}_T$ constraint [37], and the combination of the CMS and LHCb constraints on $\mathrm{BR}(B_s \rightarrow \mu ^+\mu ^-)$[38–42]. We list the parameters of the best-fit points in both the low- and the high-mass regions in Fig. 2. The overall likelihood function is quite flat in both the CMSSM and the NUHM1, so that the precise locations of the best-fit points are not very significant, and we do not quote uncertainties. This table is adapted from [36]

Model	Region	Minimum $\chi ^2$	$m_0$ (GeV)	$m_{1/2}$ (GeV)	$\tan \beta$
CMSSM	Low-mass	35.8	$670$	$1,040$	$21$
	High-mass	35.1	$5,650$	$2,100$	$51$
NUHM1	Low-mass	33.3	$470$	$1,270$	$11$
	High-mass	32.7	$1,380$	$3,420$	$39$

Comparing the current fits (solid lines and filled stars) with the results of fits to the data available in mid-2012 (dashed lines and open stars) reanalyzed with the current versions of FeynHiggs and other codes, we see that the overall extensions and shapes of the regions allowed at the 95 % CL and favoured at the 68 % CL are quite similar [36]. There is some erosion of the preferred regions at low $m_{1/2}$, due to the stronger ATLAS jets + $E{/}_T$ limit, but the most noticeable features are the shifts to larger masses of the best-fit points. However, as noted above, the differences between the values of the global $\chi ^2$ function in the low- and high-mass regions are not significant. The lower-mass regions would require less fine-tuning and hence seem more natural [73–75]. However, the interpretation of the degree of naturalness is uncertain in the absence of a more complete theoretical framework.

**Fig. 3:**
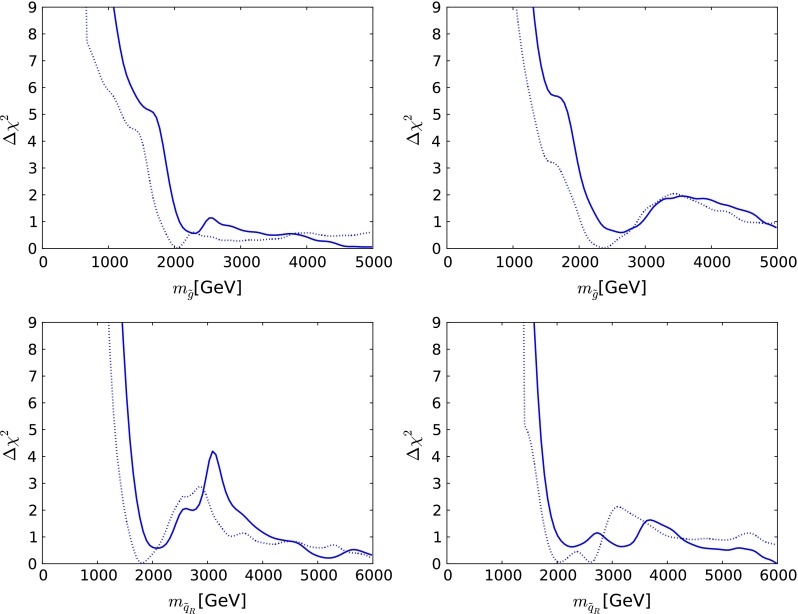
The one-dimensional $\chi ^2$ likelihood functions in the CMSSM (*left*) and the NUHM1 (*right*) for the gluino mass $m_{\tilde{g}}$ (*upper*) and a generic right-handed squark mass $m_{\tilde{q}_R}$ (*lower*) [36]. In each *panel*, the *solid line* is derived from a global analysis of the present data, and the *dotted line* is derived from an analysis if the data set used in [61], using the same implementations of the $m_h$ and dark matter scattering constraints

Figure 3 displays the one-dimensional $\chi ^2$ functions for some sparticle masses in the CMSSM (left) and the NUHM1 (right) [36]. The upper panels are for the gluino mass $m_{\tilde{g}}$, and the lower panels are for a generic right-handed squark mass $m_{\tilde{q}_R}$. The $\chi ^2$ function for $m_{\tilde{g}}$ in the CMSSM falls almost monotonically, whereas the other $\chi ^2$ functions exhibit more structure, corresponding to the structures visible in the $(m_0, m_{1/2})$ planes in Fig. 2. In each case, the $\chi ^2$ functions have been pushed up at low mass by the ATLAS jets + $E{/}_T$ limit, as seen by comparing the solid and dotted lines.

**Fig. 4:**
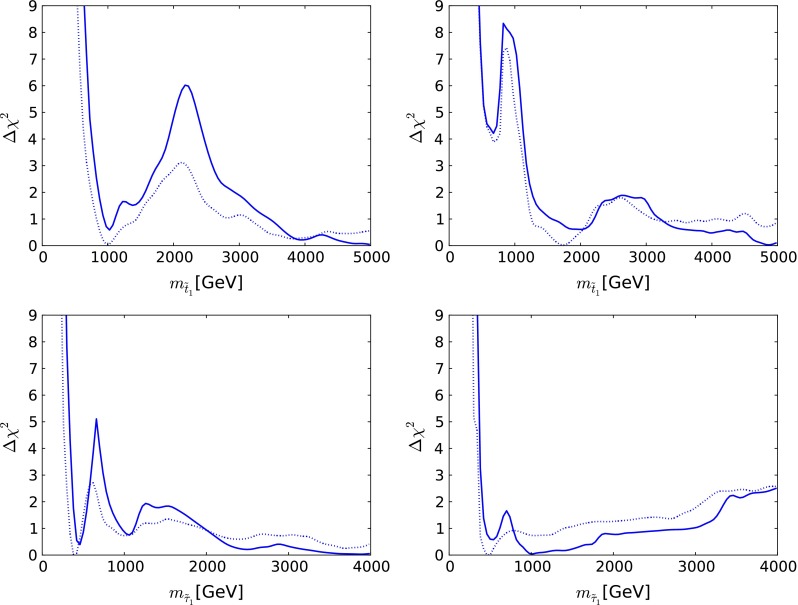
The one-dimensional $\chi ^2$ likelihood functions for $m_{\tilde{t}_1}$ (*upper*) and $m_{\tilde{\tau }_1}$ (*lower*) in the CMSSM (*left*) and the NUHM1 (*right*) [36]. In each *panel*, the *solid line* is derived from a global analysis of the present data, and the *dotted line* is derived from an analysis if the data set used in [61], using the same implementations of the $m_h$ and dark matter scattering constraints

The $\chi ^2$ function for the mass of the lighter stop squark $m_{\tilde{t}_1}$ in the CMSSM, shown in the upper left panel of Fig. 4, exhibits a local minimum at $m_{\tilde{t}_1} \sim 1{,}000$ GeV and a local maximum at $m_{\tilde{t}_1} \sim 2{,}000$ GeV [36]. On the other hand, the $\chi ^2$ function for $m_{\tilde{t}_1}$ in the NUHM1, shown in the upper right panel of Fig. 4, exhibits a local maximum at $m_{\tilde{t}_1} \sim 1{,}000$ GeV and a local minimum at $m_{\tilde{t}_1} \sim 2{,}000$ GeV, followed by another local maximum at $m_{\tilde{t}_1} \sim 2{,}600$ GeV.

The lower panels of Fig. 4 show the $\chi ^2$ functions for the lighter stau in the CMSSM (left) and the NUHM1 (right). In both cases, we see that low masses are strongly disfavoured, and that the $\chi ^2$ functions are almost flat above 1,000 GeV, with local maxima at $m_{\tilde{\tau }_1} \sim 700$ GeV.

There is no indication of a preferred supersymmetric mass scale, but one may set the following 95 % CL lower limits in GeV units [36]:

4 $$\begin{aligned} \begin{aligned}&m_{\tilde{g}} >1{,}810~\mathrm{( CMSSM)}, 1{,}920~\mathrm{( NUHM1)}, \\&m_{\tilde{q}_R} >1{,}620~\mathrm{( CMSSM)}, 1{,}710~\mathrm{( NUHM1)}, \\&m_{\tilde{t}_1} >750~\mathrm{( CMSSM)}, 1120~\mathrm{( NUHM1)}, \\&m_{\tilde{\tau }_1} >340~\mathrm{( CMSSM)}, 450~\mathrm{( NUHM1)}. \end{aligned} \end{aligned}$$

**Fig. 5:**
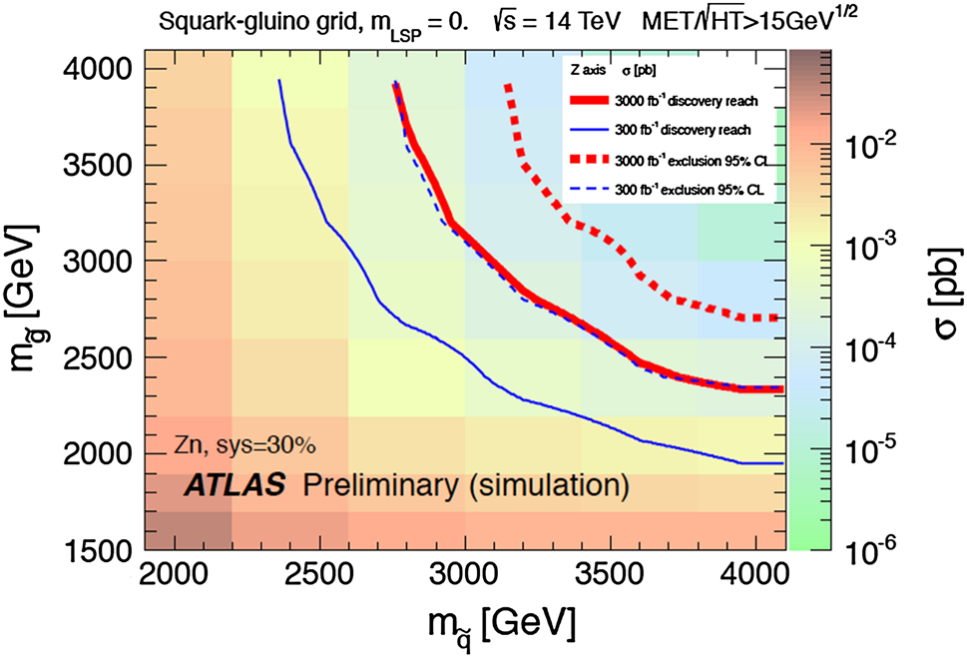
The physics reach of the LHC in the $(m_0, m_{1/2})$ plane provided by searches for squarks and gluinos assuming that the LSP mass is negligible [76]. The *different colours* represent the production cross section at 14 TeV. The *solid* (*dashed*) *lines* display the $5\sigma$ discovery reach (95 % CL exclusion limit) with 300/fb and 3,000/fb, respectively

For comparison, estimates of the supersymmetry discovery reach of the LHC with 14 TeV can be found in [76], e.g., the $(m_0, m_{1/2})$ plane displayed in Fig. 5. It was estimated in [76] that the $5\sigma$ discovery reach for squarks and gluinos with 300/fb of high-energy luminosity should be to $m_{\tilde{g}} \sim 3,500$ GeV and $m_{\tilde{q}_R} \sim 2{,}000$ GeV if $m_\chi \ll m_{\tilde{g}}, m_{\tilde{q}_R}$, and similar sensitivities are expected in the CMSSM and the NUHM1. The discovery range with 3,000/fb of luminosity would extend a few hundred GeV further, so large parts of the CMSSM and NUHM1 parameter spaces will be accessible in future runs of the LHC.

On the other hand, the lower panels in Fig. 4 and the 95 % CL lower limits on $m_{\tilde{\tau }_1}$ given in (4) suggest, within the CMSSM and NUHM1, that the lighter stau and other sleptons may lie beyond the reach of a low-energy $e^+ e^-$ collider. However, it should be emphasized that this observation is necessarily model dependent, as there is no direct information on $m_{\tilde{\tau }_1}$. If the universality assumptions of the CMSSM and the NUHM1 were to be modified appropriately, one might be able to explain the $g_\mu - 2$ discrepancy as well as offering more hope for ${\tilde{\tau }_1}$ detection in $e^+ e^-$ collisions.

**Fig. 6:**
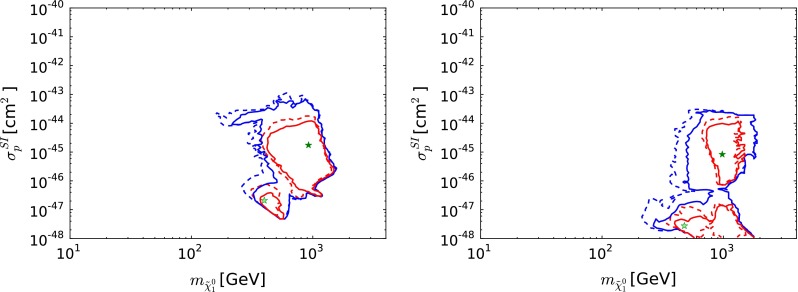
The $(m_\chi , \sigma ^\mathrm{SI}_p)$ planes in the CMSSM (*left*) and the NUHM1 (*right*) [36]. In both *panels*, the *solid lines* are derived from a global analysis of the present data, and the *dotted lines* are derived from an analysis of the data used in [61], with the current implementations of the $m_h$ and $\sigma ^\mathrm{SI}_p$ constraints. The *red lines* denote the $\Delta \chi ^2 = 2.30$ contours, the *blue lines* denote the $\Delta \chi ^2 = 5.99$ contours in each case, and the *filled* (*open*) *green stars* denote the corresponding best-fit points

Figure 6 displays the $(m_\chi , \sigma ^\mathrm{SI}_p)$ planes in the CMSSM (left) and the NUHM1 (right), again with solid (dashed) lines representing the current analysis [36] and the constraints of [61], respectively, the red (blue) lines representing 68 (95) % CL contours, respectively, with the filled (open) green stars denoting the corresponding best-fit points. We see that values of $\sigma ^\mathrm{SI}_p$ in range $10^{-47} \lesssim \sigma ^\mathrm{SI}_p\ \lesssim 10^{-43}$ cm$^2$ are allowed in the CMSSM at the 95 % CL, though the best-fit point yields $\sigma ^\mathrm{SI}_p\ \lesssim 10^{-46}$ cm$^2$. In the NUHM1, the range of $\sigma ^\mathrm{SI}_p$ preferred at the 68 and 95 % CL extends to lower values $\lesssim 10^{-48}$ cm$^2$, whilst the best-fit point yields $\sigma ^\mathrm{SI}_p\ \sim 10^{-45}$ cm$^2$, higher than the CMSSM best-fit value. These global fits indicate that $\sigma ^\mathrm{SI}_p$ may lie considerably below the current upper limit from the LUX experiment [43], though significantly above the level of the background from neutrino scattering, and hence potentially accessible to future experiments searching for the scattering of astrophysical dark matter.

There have been several claims to have observed signatures of the scattering of relatively low-mass dark matter particles, which could not be accommodated within the class of universal models discussed here. Moreover, these claims were not easy to reconcile with other negative results, e.g., from XENON100, and seem now to have been ruled out by the first results of the LUX experiment [43]. Likewise, there are various claims to have observed what might be indirect signatures of annihilations of astrophysical dark matter particles that are also difficult to accommodate within the class of models discussed here, and that will not be discussed further.

## Alternative approaches

The above results were in the CMSSM and NUHM1 frameworks, and they are quite specific to those models. This section contains some discussions of other models and proposals for model-independent analyses of LHC data.

### mSUGRA

**Fig. 7:**
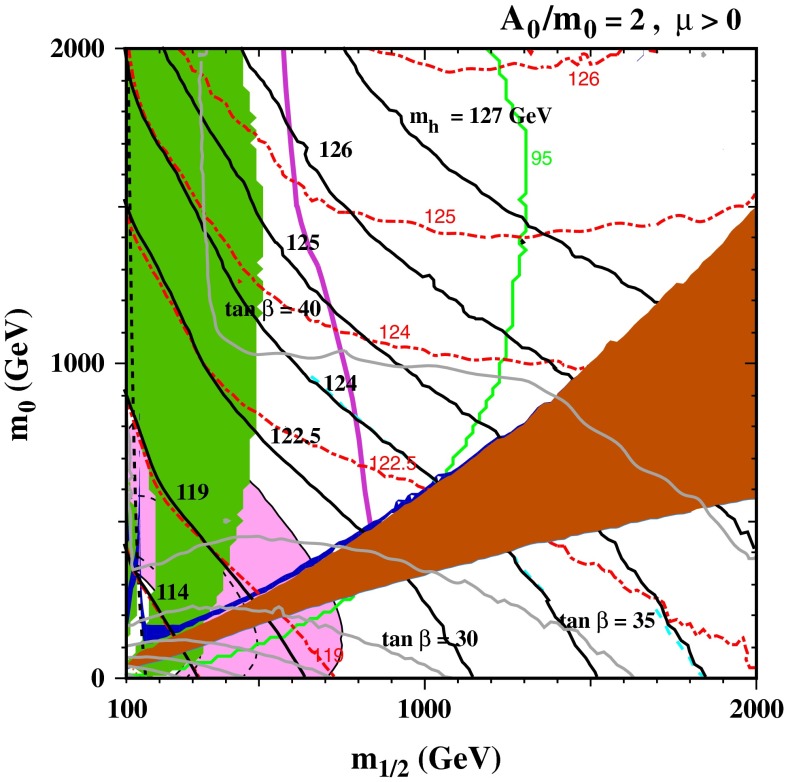
The $(m_{1/2}, m_0)$ plane in a mSUGRA model with $A_0/m_0 = 2$ [44]. In addition to the *line* and *shade* conventions used in Fig. 1, the values of $\tan \beta$ derived from the electroweak vacuum conditions are shown as *solid grey contours*

As already mentioned, mSUGRA is a more restrictive framework than the CMSSM, since the gravitino mass is equal to the scalar mass: $m_{3/2} = m_0$, and the trilinear and bilinear soft supersymmetry-breaking parameters are related: $A_0 = B_0 + m_0$. The former relation restricts the part of the $(m_{1/2}, m_0)$ plane in which the lightest neutralino is the LSP, and the second relation allows the value of $\tan \beta$ to be fixed at each point in the $(m_{1/2}, m_0)$ plane by the electroweak vacuum conditions. Figure 7 displays a typical mSUGRA $(m_{1/2}, m_0)$ plane for the particular choice $A_0/m_0 = 2$ [44]. The same conventions as in Fig. 1 are used to represent the experimental and cosmological density constraints, and the grey lines are contours of $\tan \beta$. There is a (brown) wedge of the plane where the LSP is the lighter stau, flanked by a neutralino LSP region at larger $m_0 = m_{3/2}$ and a gravitino LSP region at smaller $m_0 = m_{3/2}$. The ATLAS $E{/}_T$ search is directly applicable only in the neutralino LSP region, and would require reconsideration in the gravitino LSP region. In addition, in this region there are important astrophysical and cosmological limits on long-lived charged particles (in this case staus). The (purple) ATLAS $E{/}_T$ constraint intersects the (dark blue) dark matter coannihilation strip just above this wedge where $m_{1/2} \sim 850$ GeV, and the (green) BR($B_s \rightarrow \mu ^+ \mu ^-$) constraint intersects the coannihilation strip at $m_{1/2} \sim 1{,}050$ GeV. The portion of the coannihilation strip between this value and its tip at $m_{1/2} \sim 1{,}250$ GeV is consistent with all the constraints. In particular, in this section of the coannihilation strip the nominal value of $m_h$ provided by FeynHiggs 2.10.0 is $\in (124, 125)$ GeV, compatible with the experimental measurement within the theoretical uncertainties due to the 1–2 GeV shift in $m_h$ found in the new version of FeynHiggs, whereas the previous version would have given $m_h < 124$ GeV.

### ‘Natural’ models

**Fig. 8:**
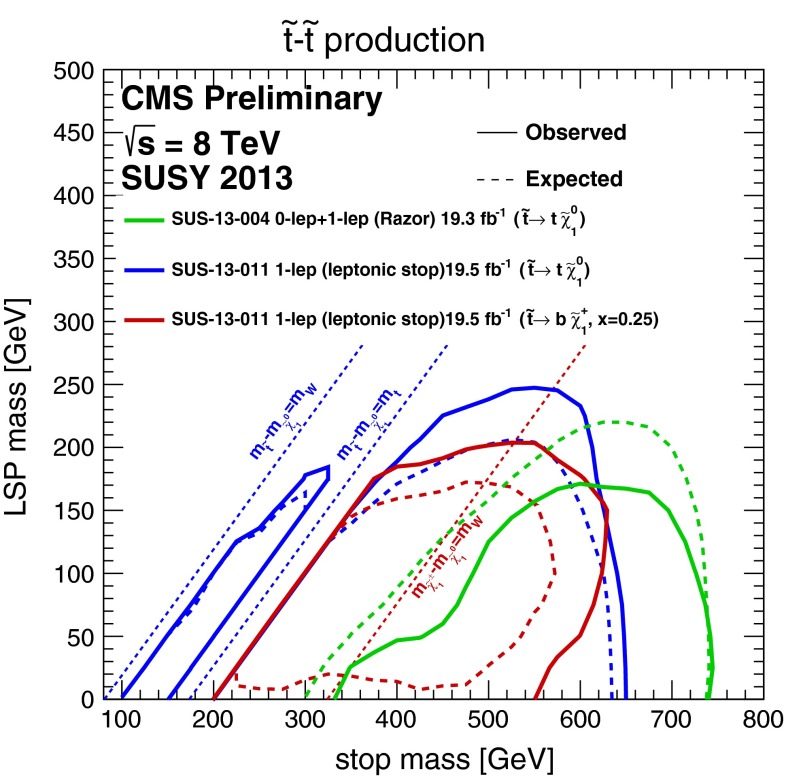
Exclusion limits from stop searches by the CMS Collaboration [77]

In view of the absence of supersymmetry in conventional jets + $E{/}_T$ searches, the fact that the lighter stop squark ${\tilde{t}_1}$ is lighter than first- and second-generation squarks in many models (as we saw earlier in the cases of the CMSSM and the NUHM1), and the fact that the naturalness (or fine-tuning) argument applies most strongly to the stop, there have been many studies of so-called ‘natural’ models in which it is assumed that $m_{\tilde{t}_1} \ll m_{\tilde{q}_R}, m_{\tilde{g}}$. Figure 8 summarizes the results of dedicated stop searches by the CMS Collaboration [77]. We see explicitly that the sensitivity of search depends on the stop decay mode assumed as well as the LSP mass assumed, and we should recall that in a realistic model stop decays may not be dominated by a single mode. So far, the dedicated stop searches do not impinge significantly on the parameter spaces of the CMSSM and the NUHM1, but this may change in the future.

### Simplified models

**Fig. 9:**
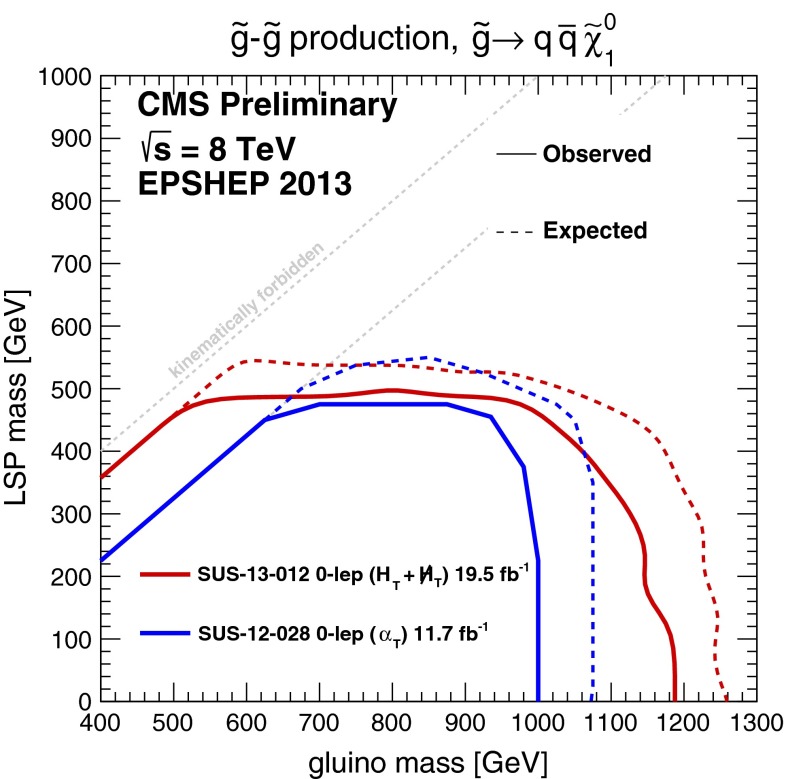
Exclusion limits from searches by the CMS Collaboration in the simplified model topology ${\tilde{g}} {\tilde{g}} \rightarrow {\bar{q}} q {\bar{q}} q \chi \chi$ [77]

Another approach has been to benchmark supersymmetric searches by assuming simplified models in which some specific cascade signature is assumed to dominate sparticle production and decay at the LHC. For example, it might be assumed that the gluinos are much lighter than all the squarks and decay dominantly into ${\bar{q}} q \chi$ final states. Figure 9 shows the exclusion limits obtained by the CMS Collaboration from a search for pair-production of gluinos in this heavy-squark limit followed by decays into ${\bar{q}} q \chi$ final states with 100 % branching ratios [77]. We see that this search also does not reach the 95 % CL lower limits in the CMSSM and the NUHM1 that were discussed earlier. We also note that such simplified models are in general over-simplified, in that typical branching ratios are $<$100 %, on the one hand, and realistic models may be tackled simultaneously using several signatures in parallel. A possible way forward building on the simplified model approach may be to parameterize a realistic model in terms of the probabilities with which specific model signatures occur and combine different signatures with a ‘mix and match’ approach to obtain the overall sensitivity to that model [78].

### Combining searches

**Fig. 10:**
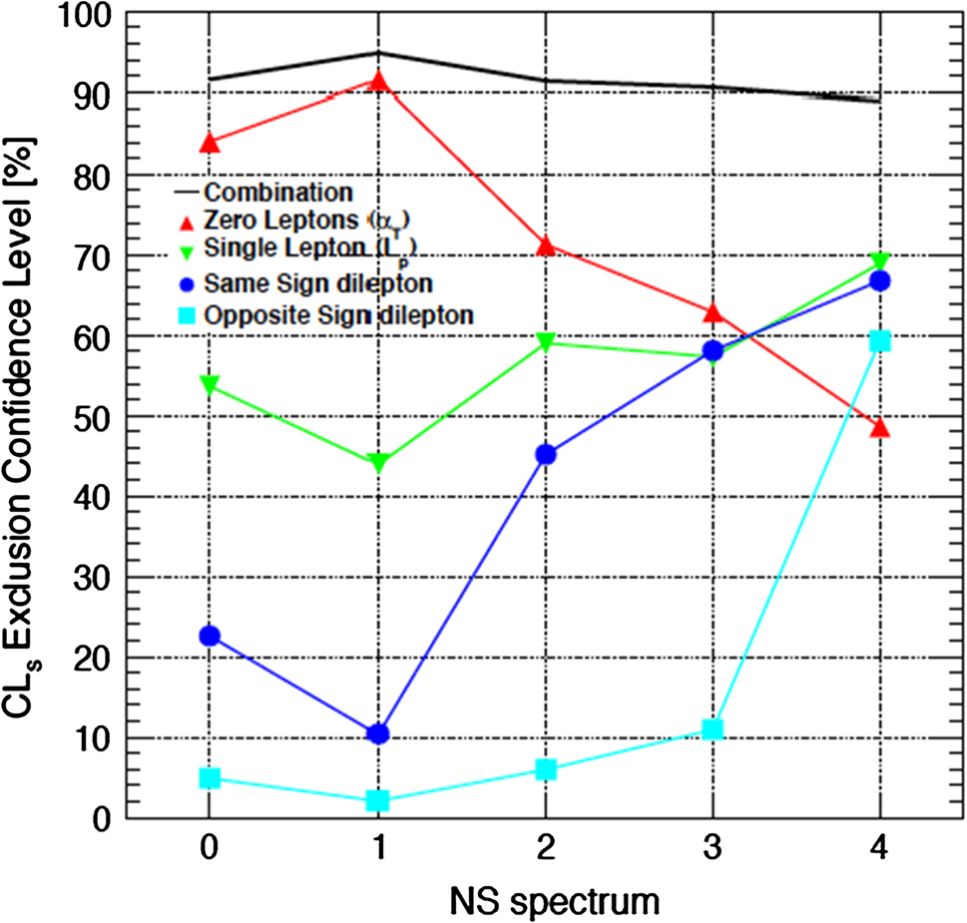
The confidence levels for excluding a class of ‘natural-like’ supersymmetric models by combining searches at 7 TeV for several different topologies: $E{/}_T$ + jets without leptons, with a single lepton and with same- and opposite-sign dileptons [79]

An interesting step in this direction was taken in [79], where it was shown that certain combinations of searches yield a sensitivity to a class of models that is almost independent of the specific parameters of the model within that class. The idea here was to combine searches for $E{/}_T$ + jets without leptons, with a single lepton and with same- and opposite-sign dileptons, and apply them to a class of ‘natural-like’ supersymmetric spectra. As can be seen in Fig. 10 where this approach was applied to 7 TeV data, the confidence level with which a particular set of gluino, third-generation squark and LSP masses ($m_{\tilde{g}} = 1$ TeV, $m_{\tilde{q}_3} = 700$ GeV, $m_\chi = 100$ GeV) could be excluded was found to be essentially independent of other details of the spectrum and associated branching ratios.

### Monojet searches

In all the above searches, the production and cascade decays of heavier supersymmetric particles were considered. A different approach, which aims to be more model independent, is to look directly for pair-production of LSPs $\chi$ with the signature of an accompanying monojet (due predominantly to initial-state gluon radiation) or electroweak boson ($\gamma$, $W^\pm$ or $Z^0$). The idea was to use such searches to constrain higher-dimensional operators that could also mediate the scattering of astrophysical dark matter. In particular, it was hoped that this approach would clarify the confusion that existed for a long time about possible experimental hints for low-mass cold dark matter particles.

**Fig. 11:**
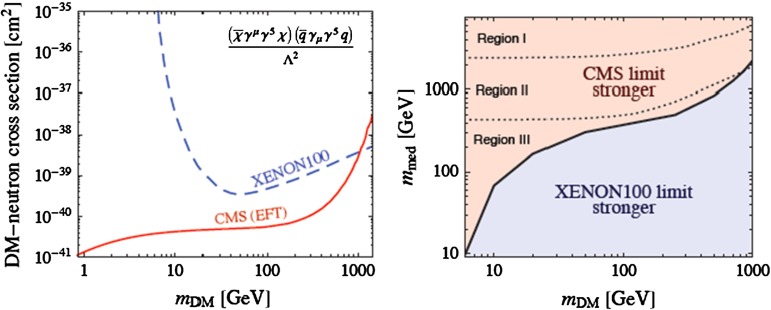
It is shown in the *left panel* that, in the effective field theory (EFT) approximation, monojet searches are more sensitive than the XENON100 search for a spin-dependent dimension-6 interaction of the form $({\bar{\chi }} \gamma _\mu \gamma _5 \chi )({\bar{q}} \gamma _\mu \gamma _5 q)/\Lambda ^2$. However, the *right panel* shows that this conclusion depends on the mass controlling the form factor of the dimension-6 interaction [80]

This approach looks promising for the case of spin-dependent dark matter scattering via an effective dimension-6 operator of the form $({\bar{\chi }} \gamma _\mu \gamma _5 \chi )({\bar{q}} \gamma _\mu \gamma _5 q)/\Lambda ^2$, as seen in the left panel of Fig. 11 [80]. However, one should remember that the kinematics of dark matter scattering (which has a very small space-like momentum transfer) and pair-production (where the momentum transfer is time-like and $>\!4 m_\chi ^2$). This raises the possibility that there may be a non-trivial form factor for the effective operator, which could suppress the sensitivity in the LHC searches for monojets, etc.. The right panel of Fig. 11 illustrates the potential importance of this effect. Whereas the LHC limit appears stronger than the XENON100 limit in the effective field theory (EFT) limit (left panel), we see that the XENON100 limit may actually be stronger, depending on the details of the theory underlying the EFT model [80]. That said, this approach is an interesting supplement to more conventional $E{/}_T$ + jets searches, and may play an increasingly important rôle in searches for supersymmetry and other new physics when the LHC restarts at high energy.

## Summary and prospects

The first run of the LHC leaves a bittersweet taste in the mouths of high-energy physicists. On the one hand, the ATLAS and CMS Collaborations have discovered a Higgs boson, an experimental Holy Grail since it was first postulated in 1964. On the other hand, they have found no trace of any other new physics, in particular no sign of supersymmetry. However, the appearance of an apparently elementary Higgs boson poses severe problems of naturalness and fine-tuning, so theorists should rejoice that they have new challenges to meet. Supersymmetry still seems to the present author to be the most promising framework for responding to these challenges, and I argue that the LHC measurements of the low mass and standard model-like couplings of the Higgs boson provide additional circumstantial arguments for supersymmetry.

The LHC searches for supersymmetry, the Higgs mass, the measurement of $\mathrm{BR}(B_s \rightarrow \mu ^+\mu ^-)$ and other experiments, notably those on dark matter, can be combined in global fits to the parameters of specific supersymmetric models [36, 62–69]. The two examples discussed here are the CMSSM and the NUHM1: analyzing models with more parameters in an equally thorough way would be far more computationally intensive. Results of global fits to the CMSSM and the NUHM1, including best-fit points, regions preferred at the 68 % CL and allowed at the 95 % CL have been presented in this paper, as well as 95 % CL lower limits on some sparticle masses. Within these models, there are reasonable prospects for discovering supersymmetry at the LHC at higher energy, as well as for observing the scattering of astrophysical dark matter.

Various alternative approaches to supersymmetry phenomenology have also been discussed, including ‘natural’ models, simplified models, combined analyses of benchmark signatures, and searches for monoboson events. Although none of these impinges significantly on the CMSSM and NUHM1 parameter spaces, all of them are likely to play greater rôles in future studies of supersymmetry at the LHC at higher energies, particularly as interest broadens to a wider range of models.

We await with impatience the advent of high-energy LHC running with increasing luminosity.

## Footnotes

1 This upper limit appears because the quartic Higgs coupling $\lambda$ is fixed in the MSSM to be equal to the square of the electroweak gauge coupling, up to numerical factors.

2 Results for the CMSSM with $\mu < 0$ can be found in [36].
